# Predictive Parameters in Patients Undergoing Percutaneous Hepatic Perfusion with Melphalan for Unresectable Liver Metastases from Uveal Melanoma: A Retrospective Pooled Analysis

**DOI:** 10.1007/s00270-022-03225-9

**Published:** 2022-08-03

**Authors:** T. M. L. Tong, M. Samim, E. Kapiteijn, T. S. Meijer, F. M. Speetjens, R. Brüning, T. H. Schroeder, S. El-Sanosy, H. Maschke, F. K. Wacker, A. Vogel, C. L. A. Dewald, J. J. Goeman, M. C. Burgmans

**Affiliations:** 1 Department of Radiology, C2-S, Leiden University Medical Center, Albinusdreef 2, 2333 ZA Leiden, The Netherlands; 2 Department of Radiology and Nuclear Medicine, University Medical Centre Utrecht, Heidelberglaan 100, 3508 GA Utrecht, The Netherlands; 3 Department of Medical Oncology, Leiden University Medical Center, Albinusdreef 2, 2333 ZA Leiden, The Netherlands; 4 Department of Radiology and Neuroradiology, Asklepios Klinik Barmbek, Hamburg, Germany; 5 Institute for Diagnostic and Interventional Radiology, Hannover Medical School, Hannover, Germany; 6 Department of Gastroenterology, Hepatology and Endocrinology, Hannover Medical School, Hannover, Germany; 7 Department of Biomedical Data Sciences, Leiden University Medical Center, Albinusdreef 2, 2333 ZA Leiden, The Netherlands

**Keywords:** Uveal melanoma, Metastases, Percutaneous hepatic perfusion, Melphalan

## Abstract

**Purpose:**

The aim of this study was to identify positive predictors for survival in uveal melanoma (UM) patients treated with percutaneous hepatic perfusion with melphalan (M-PHP), by retrospectively pooling data from three centers.

**Materials and Methods:**

Retrospective analysis including patients ($\ge$ 18 years) treated with M-PHP between February 2014 and December 2019 for unresectable liver-dominant or liver-only metastases from UM. Predictors for OS were assessed using uni- and multivariate analyses. Other study outcome measures were response rate, progression-free survival (PFS), liver progression-free survival (LPFS), overall survival (OS) and complications according to CTCAEv5.0.

**Results:**

In total, 101 patients (47.5% males; median age 59.0 years) completed a minimum of one M-PHP. At a median follow-up time of 15.0 months, complete response (CR), partial response (PR), stable disease (SD) and progressive disease were seen in five (5.0%), 55 (54.5%), 30 (29.7%) and 11 (10.9%) patients, respectively, leading to a 89.1% disease control rate. Median PFS, LPFS and OS were 9.0, 11.0 and 20.0 months, respectively. Survival analyses stratified for radiological response demonstrated significant improved survival in patients with CR or PR and SD category. Treatment of the primary tumor with radiotherapy, ≥ 2 M-PHP and lactate dehydrogenase (LDH) < 248 U/L were correlated with improved OS. Thirty-day mortality was 1.1% (*n* = 2). Most common complication was hematological toxicity (self-limiting in most cases).

**Conclusion:**

M-PHP is safe and effective in patients with UM liver metastases. Achieving CR, PR or SD is associated with improved survival. Primary tumor treatment with radiotherapy, normal baseline LDH and > 1 M-PHP cycles are associated with improved OS.

## Introduction

Uveal melanoma (UM) is the most common intra-ocular malignant tumor in adults [1]. Up to 50% of patients will develop metastatic disease with the liver being the primary predilection site [2, 3]. Without treatment, metastasized UM has a poor prognosis with reported survival rates of two to nine months after diagnosis [4]. Less than 10% of patients are eligible for resection or thermal ablation, as metastases are often bilobar and diffuse [4, 5]. The efficacy of systemic therapies is limited, except for the new immunotherapeutic drug tebentafusp [6] and some good responders in series with ipilimumab/nivolumab combination [7–9].

Two meta-analyses demonstrated that liver-directed therapies prolong OS and progression-free survival (PFS) when compared to systemic therapy [4, 10–12]. Percutaneous hepatic perfusion with melphalan (M-PHP) is a liver-directed therapy that allows administration of a high dose of melphalan directly to liver metastases with limited systemic exposure [13].

There has been emerging evidence of the efficacy of M-PHP for UM patients in recent years, and it has become a relatively established technique for the treatment of UM liver metastases. Despite the experience gained over the years with M-PHP, studies performed up until now evaluated the outcomes in small populations, partially because of the rarity of UM. Most studies are single-center cohort studies with a small sample size and focused on the efficacy and safety of M-PHP [14–23]. Limited data are available on predictive factors that may help to select patients that are most likely to benefit from M-PHP.

In this study, we pooled (partly previously published) data from different centers in Europe with the aim to evaluate efficacy and safety of M-PHP in a large cohort of UM patients. Pooling the data in this retrospective analysis allowed us to identify positive predictive factors for survival.

## Materials and Methods

### Study Design

A retrospective analysis of multicenter case series was conducted at three European centers, one in the Netherlands and two in Germany. The study was approved by the medical research ethics committees of the participating centers. Informed consent was waived for this retrospective study. All patients gave informed consent to undergo treatment.

### Patient Selection

Between February 2014 and December 2019, 103 patients were found eligible for treatment with M-PHP for UM liver metastases. Of these, 101 patients completed a minimum of one M-PHP procedure and were included in the analyses. All patients were discussed in a multidisciplinary tumor board prior to treatment. Patients eligible for treatment were $\ge$ 18 years of age with unresectable UM liver metastases. In the study centers, differences existed with regard to selection of patients with limited extrahepatic disease: these were not considered eligible for M-PHP at the Dutch center. In one German center, extrahepatic disease was not an exclusion criterium and at the other center patients with extrahepatic metastases $>$ 10 mm were excluded.

As mentioned above, in two patients the M-PHP treatment could not be completed and the data of these patients were not used for the analysis. In one patient, M-PHP was stopped due to blood clots in the chemofilters during two separate attempts. In another patient, ECG changes suggestive of cardiac ischemia occurred after occlusion of the caval vein. The procedure was stopped and the patient recovered without sequelae. The results of part of the study population (74 patients) presented in this study have been published previously in single-center analyses [20, 22, 24–26].

### M-PHP Procedure

The M-PHP procedure has been extensively described previously [14, 17, 25, 27]. A short description is provided in “Appendix”. Ideally, a minimum of two M-PHPs was performed with a melphalan dose of 3 mg/kg (maximum 220 mg). Treatment was discontinued in case of progressive disease after the first M-PHP or intolerance to treatment. If no disease progression occurred, a second M-PHP was performed between 6 and 10 weeks after the first procedure. More than two M-PHPs were performed in individual cases if patients had progressive hepatic disease and were considered eligible for repeated treatment with M-PHP.

### Outcome Measures

Study outcome measures were response rate (RR), PFS, liver progression-free survival (LPFS), OS and safety. Furthermore, predictors for prolonged OS were analyzed.

### Response Assessment

Response was evaluated according to Response Evaluation Criteria in Solid Tumors 1.1 (RECIST 1.1) and defined as either progressive disease (PD), stable disease (SD), partial response (PR) or complete response (CR). Objective response rate (ORR) and disease control rate (DCR) were defined as the percentage of patients with ‘PR or CR’ and ‘SD, PR or CR,’ respectively. The interval between M-PHP and first follow-up imaging differed between participating centers and was an average of seven, three and eight weeks. After this, all patients received consecutive follow-up imaging every 3–4 months until disease progression or death.

### Survival

PFS and LPFS were measured as the time interval from date of first M-PHP until overall or intrahepatic progression, respectively, or death, whichever occurred first. OS was the time interval measured from date of first M-PHP until last follow-up or death, whichever occurred first.

### Adverse Events

All recorded procedure-related clinical and hematological adverse events (AEs) within 30 days after M-PHP were described according to Common Terminology Criteria for Adverse Events version 5.0 (CTCAEv5.0). All procedures included in safety analyses were performed as part of the first M-PHP treatment cycle. Twenty-two patients underwent repeated M-PHP after the first treatment cycle (mean 1.3, range 1–3) for recurrence after an initial good response to M-PHP. These additional treatments (*n* = 29) were not included in the safety analyses.

### Statistical Analysis

Statistical analyses were performed using SPSS 25.0 (SPSS Inc., Chicago, IL, USA) and R version 3.1.2 open-source software. Descriptive statistics were used for baseline characteristics. The Kaplan–Meier method was applied to analyze PFS, LPFS and OS including 95% confidence intervals (95% CI). Patients who were lost to follow-up were censored in the survival analysis. OS was stratified according to response group: ‘CR and PR,’ ‘SD’ or ‘PD.’ The log-rank test was used to compare curves. Uni- and multivariable analyses (UVA and MVA) were performed with RStudio using the Cox proportional hazards model to determine possible independent predictors for OS. The proportional hazard assumption was checked with a test based on residuals (Schoenfeld’s global test). In the UVA and MVA, the covariate effect was estimated while adjusting for between-center heterogeneity. In UVA and MVA, the models were fitted using a general estimating equation (GEE) approach to account for between-center effect [28]. UVA was performed based on clinically relevant variables. In the MVA, statistically significant variables according to the UVA as well as clinically relevant variables were incorporated. For the regression analyses, missing data were imputed by multiple imputation using the predictive mean matching method. A *p* value < 0.05 was considered as statistically significant. The adverse event data were presented based on total number of procedures; the survival analyses were patient-based.

## Results

### Patient and Procedure-Related Characteristics

Baseline characteristics of the 101 patients [47.5% males; median age 59.0 years (range 38–83)] are presented in Table 1. These 101 patients underwent a total of 212 M-PHP procedures (median 2, range 1–5). Seventy-seven (76.2%) patients underwent at least two M-PHP procedures. Twenty-five patients (24.8%) received more than two M-PHPs. Mean administered melphalan dose for the first and second M-PHP was 196.9 mg (range 108.0–223.5) and 188.2 mg (range 110.0–223.5), respectively. There was a median interval of 8 weeks (range 5.0–34.0 weeks) between first and second M-PHP.

**Table 1:** Baseline patient characteristics

	*N*	%
Number of patients	101	
Center 1	62	61.4
Center 2	20	19.8
Center 3	19	18.8
Gender		
Male	48	47.5
Female	53	52.5
Age [median (range)]	59.0 (38 to 83)	
Length [median (range)]	172.0 (157 to 195)	
Weight [median (range)]	76.0 (51.7 to 117)	
Interval primary tumor to metastases [median, months (range)]	28.0 (− 1 to 232)	
Treatment primary tumor		
Enucleation	41	40.6
Radiotherapy	41	40.6
Unknown	19	18.8
Type of metastases		
Synchronous	12	11.9
Metachronous	89	88.1
Number of metastases		
1–5	28	27.7
6–9	22	21.8
> 9	51	50.5
Mutation status liver metastases		
GNA11	17	16.8
GNAQ	30	29.7
Missing	54	53.5
Prior therapy liver metastases		
Regional	19	18.8
Systemic	10	9.9
Regional and systemic	4	4.0
None	66	65.3
Unknown	2	2.0
Type of lesion		
Hypervascular	67	66.3
Hypovascular	19	18.8
Mixed	15	14.9
Number of M-PHP procedures		
1 M-PHP	24	23.8
> 1 M-PHP*	77	76.2
> 2 M-PHP*	25	24.8
Extrahepatic metastases at baseline	7	6.9
LDH [median (range)]	228 (123–4608)	
LDH normal	54	53.5
LDH < 2× ULN	29	28.7
LDH > 2× ULN	11	10.9
Unknown	7	6.9

*LDH* lactate dehydrogenase, *ULN* upper limit of normal

*The number of patients with > 2 M-PHP procedures is also included in the number of patients with > 1 M-PHP procedure

### Response

After a median follow-up time of 15.0 months, CR was achieved in 5 patients (5.0%), PR in 55 (54.5%) and SD in 30 (29.7%), resulting in an ORR of 59.4% and DCR of 89.1%. Eleven patients (10.9%) experienced PD.

### Survival

Time to death was unknown in twelve patients that were lost to follow-up. No trend was detected in the lost to follow-up group in terms of radiological response, and these patients were censored in the OS analysis. Median PFS, LPFS and OS were 9.0 months (95% CI 7.7–10.3), 11.0 months (95% CI 9.0–13.0) and 20.0 months (95% CI 13.7–26.3), respectively.

**Fig. 1:**
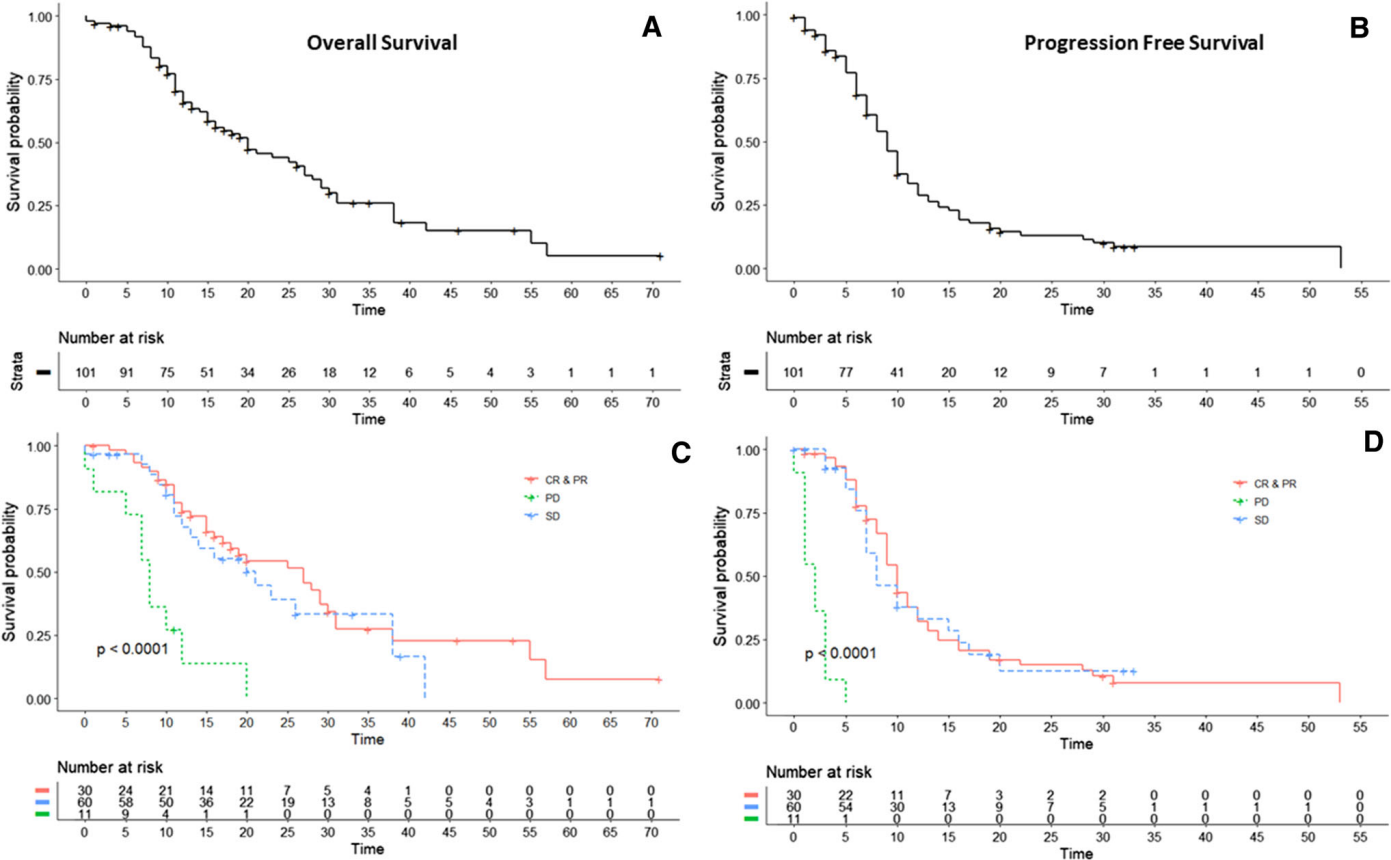
Kaplan–Meier curves for OS (**A**, **C**) and PFS (**B**, **D**). The median OS and PFS were 20 months (95% CI 13.7–26.3) and 9.0 months (95% CI 7.7–10.3). In **C**, **D**, the OS and PFS are stratified according to response category

Median PFS was 10 months (95% CI 8.9–11.1), 8 months (95% CI 5.1–10.8) and 2 months (95% CI 1.0–3.1) for the ‘CR and PR,’ ‘SD’ and ‘PD’ groups, respectively. The median LPFS was 12 months (95% CI 10.1–13.9), 12 months (95% CI 6.1–17.9) and 5 months (95% CI 1.2–8.8) for the ‘CR and PR,’ ‘SD’ and ‘PD’ group, respectively. Median OS was 27 months (95% CI 17.5–36.5) for the ‘CR and PR’ group, 21 months (95% CI 11.2–30.8) for the ‘SD’ group and 8 months (95% CI 5.7–10.3) for the ‘PD’ group. The difference between the response groups was statistically significant (*p* < 0.001) (Fig. 1).

Subgroup analyses were performed to evaluate the effect of procedure number on survival. Median PFS and LPFS was, respectively, 9 versus 8 months and 11 versus 9 months for patients treated with $\ge$ 2 M-PHPs compared to patients treated with one M-PHP. This difference was not statistically significant. A statistically significant difference in OS was found for $\ge$ 2 M-PHP treatments versus one treatment (20 versus 8 months, respectively, *p* < 0.05).

### Regression Analyses

In UVA, a larger sum of target lesions, only one M-PHP procedure (compared with ≥ 2 M-PHP procedures) and lactate dehydrogenase (LDH) levels > 248 U/L were correlated with poor OS. In MVA, treatment of the primary tumor with radiotherapy (compared to enucleation) as well as $\ge$ 2 M-PHP procedures was associated with improved OS, while elevated LDH levels (> 248 U/L) at baseline remained an independent predictor of worse OS (Tables 2, 3).

**Table 2:** Univariable analysis for overall survival

	HR	95% CI	*p* value
Age*	0.998	0.962–1.036	0.915
Gender (female)	0.916	0.675–1.243	0.571
Treatment primary tumor			
Enucleation	–	–	–
Radiotherapy	0.933	0.763–1.140	0.497
Vascularity liver metastases			
Hypervascular	–	–	–
Hypovascular	0.852	0.434–1.673	0.642
Mixed	1.064	0.540–2.094	0.858
Previous treatment liver metastases			
None	–	–	–
Systemic	1.180	0.520–2.680	0.693
Local	1.470	0.944–2.287	0.088
Combination	0.496	0.161–1.533	0.223
Number liver metastases			
< 5	–	–	–
6–9	1.135	0.527–2.444	0.747
> 9	1.526	0.855–2.723	0.153
Sum target lesions*	1.037	1.008–1.067	0.013
Number M-PHP procedures > 1	0.450	0.224–0.905	0.025
Interval primary to liver (months)*	0.996	0.987–1.005	0.404
LDH at baseline			
Normal**	–	–	–
< 2× ULN	1.424	1.245–1.630	< 0.001
> 2× ULN	3.687	1.370–9.922	0.010

*Continuous variable

**Normal LDH value: < 248 U/L

**Table 3:** Multivariable analysis for overall survival

	HR	95% CI	*p*
Age*	0.998	0.974–1.023	0.898
Treatment primary tumor			
Enucleation	–	–	–
Radiotherapy	0.672	0.530–0.852	0.001
Sum target lesions*	1.024	0.976–1.075	0.330
Number M-PHP procedures > 1	0.493	0.247–0.984	0.045
LDH at baseline			
Normal**	–	–	–
< 2× ULN	1.308	1.063–1.610	0.011
> 2× ULN	3.110	1.279–7.559	0.012

*Continuous variable

**Normal LDH value: < 248 U/L

### Safety

In total, 183 M-PHPs were analyzed on safety according to CTCAEv5.0.

Peri-procedural complications included dissections (*n* = 6) or occlusion (*n* = 1) of the hepatic artery, clot formation in the extracorporeal filtration circuit (*n* = 2), atrial fibrillation with cardioversion, balloon leakage, vaginal hemorrhage and neck hematoma (*n* = 1 each). One patient developed hypothermia and metabolic acidosis and was observed for one night in the Post-Anesthesia Care Unit, and one patient was transferred to the intensive care due to hemodynamic instability and decreased saturations; both patients made a full recovery.

An overview of post-procedural complications is provided in Table 4. The most common post-procedural complication was hematological toxicity. This was of low-grade (1/2) and self-limiting in the majority of patients. The most frequent clinically relevant post-procedural AEs were thromboembolic complications: pulmonary embolism and strokes each occurred after five M-PHPs (Table 4). One patient developed a NSTEMI post-procedurally. Post-procedural mortality within 30-day was 1.1%. One patient died 3 days after M-PHP from toxic liver failure, generalized bleeding due to coagulopathy and lactate acidosis. A second patient passed away 12 days after the procedure due to rapid tumor progression and subsequent progressive multiorgan failure.

**Table 4:** Post-procedural adverse events according to CTCAE v 5.0 based on 183 M-PHP procedures

Adverse events	Grade 3 [*n* (%)]	Grade 4 [*n*(%)]	Grade 5 [*n*(%)]
Hematological			
Anemia	13 (7.1)	1 (0.5)	
Leucopenia	16 (8.7)	28 (15.3)	
Thrombocytopenia	26 (14.2)	26 (14.2)	
Hepatic			
Increased AST	12 (6.6)	2 (1.1)	
Increased ALT	10 (5.4)	2 (1.1)	
Increased bilirubin	4 (2.2)		
Gastrointestinal			
Gastric ulcer	1 (0.5)		
Vascular			
Pulmonary embolism	5 (2.7)		
Cardiac			
Cardiac ischemia	1 (0.5)		
Metabolism and nutrition			
Hyperglycemia	1 (0.5)		
Tumor lysis syndrome	1 (0.5)		
Renal/urinary			
Acute kidney injury	3 (1.6)		
Infections			
Vulvar infection	1 (0.5)		
Sepsis		1 (0.5)	
Febrile neutropenia	5 (2.7)		
Others	1 (0.5)		
Nervous system			
Vasovagal reaction	2 (1.1)		
Stroke	5 (2.7)		
General disorders			
Fatigue	1 (0.5)		
Death			2 (1.1)

*AST* aspartate aminotransferase, *ALT* alanine aminotransferase

## Discussion

Our study provides further evidence for the efficacy of M-PHP in the treatment of patients with UM hepatic metastases. The median OS of 20.0 months from initial treatment with M-PHP in our cohort of 101 patients is consistent with results from previous studies. Recently published series resulted in median OS ranging from 8.0 to 27.4 months [15, 17–20, 22, 24, 25, 29]. Our ORR of 59.4% is also in accordance with ORRs (range 33.3 to 72%) reported in aforementioned studies.

In this study we found that LDH > 248 U/L was a negative predictor of survival. This is in line with previous literature, describing that high LDH levels are correlated with worse survival in various cancer types [30]. Furthermore, primary tumor treatment with radiotherapy was associated with improved survival. Most likely this indicates that tumor stage at initial presentation is a predictive factor as patients eligible for treatment with radiotherapy often have a smaller primary tumor size [31]. The literature shows that tumor size and factors as chromosome 3-loss and BAP1 mutation determine the risk of metastatic disease [32]. As our study shows, patients treated with radiotherapy for their primary tumor also have superior survival outcomes once metastases have occurred and/or respond better to M-PHP. Furthermore, both UVA and MVA confirmed that patients treated with more than one M-PHP had a better survival, compared to those treated with only one M-PHP. This is related to the fact that patients who show PD after the first M-PHP do not qualify for second M-PHP. Lastly, we found that radiological response or tumor control (CR, PR and SD) is associated with superior survival. This indicates that effective treatment of liver metastases translates to overall survival benefit and confirms that patients showing PD after the first M-PHP will not have the same survival benefit as responders.

Our study confirms that M-PHP has an acceptable safety profile with mostly grade 1/2 and self-limiting toxicity. This is consistent with previous reports [23, 25]. However, serious complications may occur. In our cohort, the mortality rate was 1.1% within 30 days after M-PHP. Other studies have reported procedure-related mortality of 4.3% [15]. This warrants careful consideration whether the benefits outweigh the risks in an individual patient. Best candidates for M-PHP are fit patients with good non-cancer-related health status, no cardiovascular disease, early-stage primary UM, limited metastatic burden, liver-only disease and LDH < 2× upper limit of normal (ULN). For such patients, M-PHP is the preferred first-line therapy as response rates and survival after M-PHP are superior to those after systemic chemotherapy [33].

Recently systemic immunotherapy led to a breakthrough in the treatment of patients with metastatic cutaneous melanoma (CM) and immune checkpoint inhibitors (ICI) improved survival in these patients. Unfortunately, the efficacy of ICI in patients with UM is lower compared to CM patients [34]. Compared to CM, UM has a lower mutational burden and this may lead to poor recognition of cancer cells by T-cells. Recent developments with the immunotherapeutic agent tebentafusp, a target to the antigen gp100 that is presented by HLA-A*02:01, showed a 1-year OS of 73% compared to 59% in the control group in a phase III trial [6]. However, patients are only eligible for treatment if they are HLA-A*02:01-positive. Effective systemic therapies are still lacking for HLA-A*02:01-negative patients, but combination of ipilimumab and nivolumab seems promising in small series [7–9]. We are currently conducting a randomized phase I/II trial (NCT04283890) investigating the efficacy of M-PHP with ipilimumab and nivolumab compared to M-PHP alone [35]. Hopefully, this study will lead to further improvement of the prognosis of patients with metastatic uveal melanoma, in particular for patients with both hepatic and extrahepatic disease.

Our study has several limitations, the retrospective nature and lack of a comparative group being the most important. Furthermore, differences existed between the participating centers with regard to patient selection and follow-up timing and data. Most notably, patients with extrahepatic disease were excluded in two centers, but limited extrahepatic disease was allowed in the third center. The number of patients with extrahepatic disease in this study was too small (*n* = 7), to demonstrate a statistical difference in survival compared to patients with liver only disease. It seems likely that extrahepatic disease has a negative influence on the OS and PFS and this is supported by previously reported data [25]. While we had information on the primary tumor treatment, information on the primary tumor stage was not available for a considerable number of patients in this retrospective study. Finally, although in the statistical analysis we corrected for center effect, the problem of selection bias is not completely resolved.

## Conclusion

This study with over 100 patients with UM liver metastases confirms that M-PHP is an effective palliative treatment, with a high ORR and median OS of 20 months. Independent predictors of prolonged survival are normal baseline LDH level, radiotherapy as primary treatment (most likely reflecting lower tumor stage of the primary malignancy) and completion of at least two M-PHP procedures. Finally, we demonstrated that radiological response (CR and PR) and disease control rate (CR, PR and SD) are associated with superior survival indicating that effective treatment of the liver metastases translates to overall survival benefit.

## Appendix: Description of M-PHP Procedure

All procedures were performed in an angiographic suite, under general anesthesia. The procedure team consisted of an interventional radiologist, anesthesiologist and extracorporeal perfusionist. At the start of the procedure, an initial heparin dose of 300 U/kg was administered, and an activated clotting time of ≥ 450 s (> 500 s in two centers) was maintained throughout the procedure. Melphalan was either administered directly to the proper hepatic artery or split and infused in the right and left hepatic artery (selective lobar approach) in a dose of 3 mg/kg of body weight. The coagulation status was corrected with protamine sulfate 3 mg/kg after the procedure.

In all centers, patients received granulocyte colony-stimulating factor (G-CSF) within 72 h after M-PHP. Additionally, patients in one German center also received a single course of antibiotics post-procedurally. At the Dutch hospital, the melphalan dose was reduced to 75% of the original dose in case of grade 3/4 hematologic toxicity after the first M-PHP.
